# Efficacy and Safety of LigaSure in Laparoscopic Sutureless Appendectomy

**DOI:** 10.7759/cureus.24764

**Published:** 2022-05-05

**Authors:** Vipin Gupta, Somendra Pal Singh Chauhan, Mayank Gupta, Ramlakhan Verma, Shailendra Pal Singh, Anand Panday

**Affiliations:** 1 General Surgery, Uttar Pradesh University of Medical Sciences, Saifai, Etawah, IND; 2 Pediatric Surgery, King George’s Medical University (KGMU), Lucknow, IND

**Keywords:** appendectomy, sutureless appendectomy, ligasure, laparoscopic appendectomy, appendicitis

## Abstract

Background

The closure of the appendiceal stump is a crucial step during an appendectomy. The purpose of this study is to evaluate the LigaSure Vessel Sealing System in laparoscopic appendectomy (LA) for sealing and dividing the base of the appendix.

Material and methods

Laparoscopic appendectomy was performed using the 5-mm LigaSure Vessel Sealer in 53 patients, and the mesoappendix along with the base of the appendix was divided by LigaSure. Patient demographic details, operative time, return to oral feed, duration of hospital stay, and postoperative complications were recorded, and statistical analysis was done.

Results

Out of 53 patients (24 women and 29 men), no complications occur in 51 patients. The mean age and standard deviation (SD) were 26.50 ± 10.46 years. The mean operative time for 53 appendectomies by LigaSure was 27.8 ± 6.72 minutes. The mean duration of hospital stay after surgery was 3.3 ± 0.72 days. One patient developed mild subcutaneous emphysema over the abdomen (1.8%), and surgical site infection occurred in one patient (1.8%).

Conclusion

This study demonstrated that sealing and dividing the base of the appendix by the LigaSure Vessel Sealing System is safe and feasible. It is associated with low complication rate and may help in simplifying the operative procedure.

## Introduction

Acute appendicitis is the most common explanation of acute abdomen, which compels the patient to seek medical attention. Approximately 7% of the population will suffer from acute appendicitis in their lifetime, with the peak occurrence between the second and third decade [1]. Appendectomy is the most commonly done emergency abdominal operation worldwide. The open method of appendectomy has remained the gold standard technique for acute appendicitis for over a century due to the procedure’s low morbidity and mortality [2]. In 1982, a German gynecologist, Semm, performed the first successful laparoscopic appendectomy (LA) [3]. Laparoscopic appendectomy (LA) has advanced from the time it was first performed. Laparoscopic appendectomy has gained popularity as a diagnostic and treatment modality for acute appendicitis with technological progress over the last two to three decades. Laparoscopic appendectomy has advantages over the open approach in less postoperative pain, shorter hospital stay, faster postoperative rehabilitation, and fewer postoperative complications. There are negative issues, such as higher cost, longer duration of surgery, and a higher margin of intra-abdominal abscess [4-6].

The closure of the appendiceal stump remains the foremost crucial step during appendectomy because many of the postoperative complications are caused by its inappropriate management. The development of life-threatening conditions such as stercoral fistulas, postoperative peritonitis, and sepsis is included in these complications. Currently, two techniques are most ordinarily used for laparoscopic appendectomy: division of the mesoappendix with the harmonic scalpel (HS) and ligation of the appendix with an endoloop, and division of the mesoappendix and appendix with an endo stapler. Other methods used to close the stump in LA include intracorporeal ligation, titanium clips, handmade loops, nonabsorbable polymer clips (Hem-o-lok clips), and extracorporeal sliding knot. However, the appendicular stump closure’s preferred technique still appears to be controversial.

Staplers can divide and seal both the mesoappendix and the appendix base simultaneously. Studies have proven that it is both easy to apply and safe. One of the major advantages of staplers is they are safe even when the appendix base is inflamed, and they also enable a partial tangential resection of the cecum. One major disadvantage is that staplers are more expensive than other methods [7,8]. The downside of clip use is that it is not sound in the case of intense inflammation where the appendicular base is wide. Endoloop in various studies is a safe, cheap, and cost-effective method that is easy to construct and apply with the disadvantage of prolonging the operation time [9-11].

LigaSure is a bipolar electrosurgical instrument, which can be used for hemostasis in laparoscopic and open surgery. LigaSure was introduced in 1998; it is used for sealing blood vessels of up to 7 mm in diameter. In addition to sealing blood vessels, modern instruments can also be used to grasp and cut a variety of tissues. It consists of an electrical current generator and an instrument for grasping blood vessels. The generator produces an electrical current across the vessel wall. Electromagnetic waves energize the electrons within the vessel. These electrons release their energy as heat. The elastin and collagen found within the vessel wall denature when the vessel is heated. The generator precisely controls the quantity of energy delivered to the tissue. The majority of the generator systems monitor the impedance within the circuit, and when it begins to rise, they automatically break the circuit. This prevents the burning of the vessel wall. After a period of cooldown, the elastin and collagen form a seal. Specifically, low voltage and high current are delivered to the targeted tissue while the mechanical pressure from the instrument allows the denatured protein to make a coagulum [12,13].

This paper describes our experience of using the LigaSure Vessel Sealing System in LA to seal and divide the base of the appendicular stump without the need for any other suturing/mechanical device.

## Materials and methods

We prospectively evaluated 53 cases of laparoscopic appendectomy done using the 5-mm (LF1737 Maryland Jaw Laparoscopic Sealer) LigaSure Vessel Sealing System (Covidien ForceTriad, Mansfield, MA, USA). All the cases are operated in the Department of General Surgery, Uttar Pradesh University of Medical Sciences (UPUMS), Saifai, from January 2020 to July 2021. It was approved by the university ethical committee (116/2019-20). Patients were included in the study after proper clinical examination, laboratory findings, and ultrasonographic evidence of acute appendicitis in the outpatient department and emergency. Written informed consent was obtained from all patients participating in the study.

All patients diagnosed with uncomplicated appendicitis admitted for laparoscopic appendectomy were included in the study. Patients with findings of appendicular lump, appendicular abscess, appendicular perforation, and gangrenous appendicitis involving the base of the appendix were excluded from the study. The patients were given an intravenous antibiotic before the induction of anesthesia.

Patient demographic details, duration of surgery, return of bowel sound, return to oral feeds, intraoperative and postoperative complications, and duration of hospital stay were noted.

Continuous variables such as the age of the patient, duration of hospital stay, and duration of surgery were presented as mean ± standard deviation (SD), while categorical variables such as postoperative complication and gender were expressed as frequency and percentages.

Procedure

**Figure 1:**
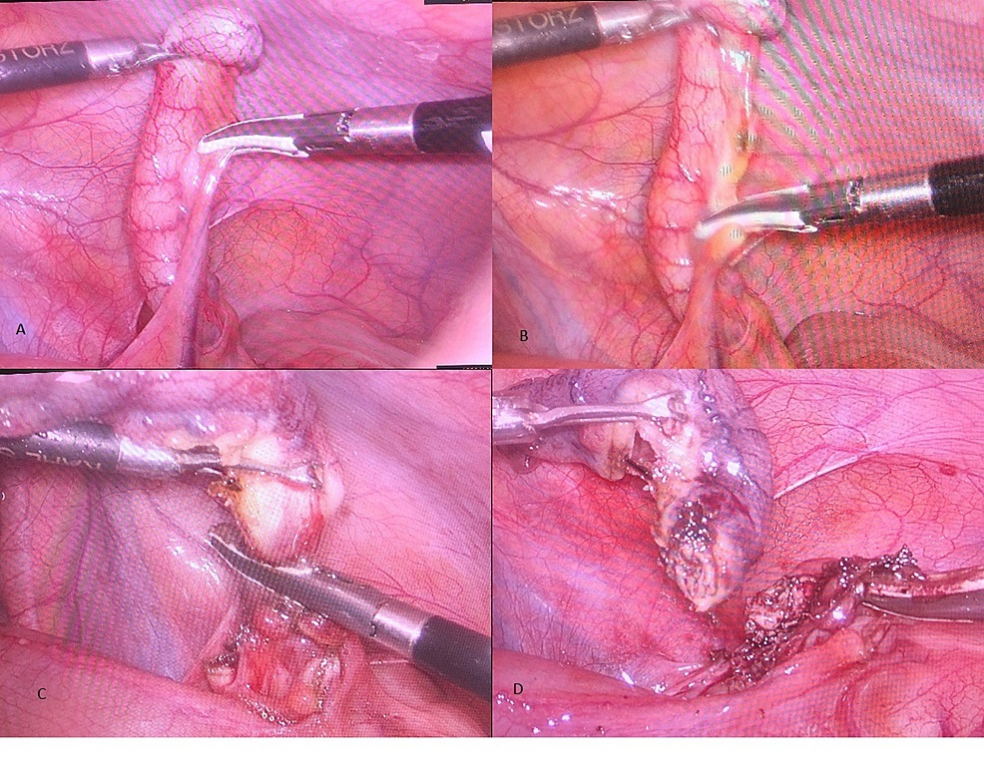
Intraoperative pictures showing the use of the 5-mm LF1737 Maryland Jaw Laparoscopic Sealer A and B: The sequential division of the mesoappendix by LigaSure. C: After the division of the mesoappendix, the appendix is coagulated and cut by the repeated application of LigaSure. D: The dissected appendix along with the appendicular stump.

The patient was placed in the supine position, along with the Trendelenburg position and left lateral position. A 10-mm infraumbilical incision was given, and a Veress needle was introduced. Pneumoperitoneum was created by insufflating carbon dioxide (CO2) gas at a pressure of 12-14 mmHg, depending on the patient’s body weight and age. A 30° telescope was inserted, and two 5-mm working ports were placed in the left lower quadrant and suprapubic region. Diagnostic laparoscopy was performed, and the appendix was identified. LigaSure was used to coagulate the mesoappendix. Thereafter, the base of the appendix was coagulated, sealed, and divided by the repeated application of LigaSure (Video 1) and retrieved through a 10-mm port. The stump was verified after appendectomy regarding complete sealing of the lumen (Figure 1).

**Video 1:** Division of the mesoappendix and appendix using the 5-mm LF1737 Maryland Jaw Laparoscopic Vessel Sealer Video credits: Prof. (Dr.) Vipin Gupta

Postoperative care and follow-up

Postoperatively, oral intake of fluids was allowed after the return of bowel sound, and patients were discharged about 24 hours after tolerating oral feed. The patients were followed for three months from the time of discharge and looked after for any complications.

## Results

Out of 53 patients, 29 (56.48%) patients were male and 24 (43.52%) patients were female. The maximum age was 60 years, and the minimum age was 11 years. The mean age and standard deviation (SD) were 26.50 ± 10.46 years (range: 11-60 years). None of our patients had any preexisting comorbid conditions such as diabetes, hypertension, or any chronic illness. Neutrophilia in routinely run laboratory tests showed a mean of 72.6% neutrophil differential, with a standard deviation of 8%, and white blood cell count had a mean of 11.4 × 10^9^/L, with a standard deviation of 2.64 × 10^9^/L. The mean operative time for 53 appendectomies by LigaSure was 27.8 ± 6.72 minutes (range: 21-50 minutes). Return of bowel sound occurs on postoperative day 1 (POD1) in 42 cases (77.35%), POD2 in eight cases, and POD3 in three cases. The mean duration of hospital stay after surgery was 3.3 ± 0.72 days.

Out of 53 appendectomies, no complications occurred in 51 cases. One patient develop mild subcutaneous emphysema over the abdomen, which was managed conservatively (1.8%), and postoperative surgical site infection occurred in one (1.8%) case, which was managed by cleaning and regular dressing. No patient underwent a conversion to an open appendectomy. Fortunately, no serious postoperative complications such as fecal fistula, abdominal abscess, postoperative peritonitis, and sepsis occur in any patient. We observed no intraoperative complications and no mortality in any patient during our study (Table 1).

**Table 1:** Patient demographic details with operative outcomes

Factor	Range
Age (years)	26.50 ± 10.46 (11-60)
Sex (male:female)	1.2:1
WBC count (10^9^/L)	11.4 ± 2.64
Operation time (minutes)	27.8 ± 6.72 (21-50)
Return of bowel sound	
POD1	42 (77.35%)
POD2	8 (15%)
POD3	3 (5.6%)
Return to oral feed	2.3 ± 0.69
Length of hospital stay after surgery (days)	3.3 ± 0.72
Postoperative complications	2/53 (3.7%)
Subcutaneous emphysema over the abdomen	1/53 (1.8%)
Surgical site infection	1/53 (1.8%)

## Discussion

LigaSure has been used widely in many surgical approaches in several fields including gynecology, gastrointestinal system, endocrinology, and urology. There are no clinical reports of LigaSure usage in laparoscopic appendectomy; however, experimental studies have shown it to be of use. There may be concerns about incomplete closure of the appendiceal lumen by a nonmechanical device; however, it is shown from our previous study using a harmonic scalpel (HS) that such an instrument can be successful without any mechanical closure of the lumen [14]. Although we had not compared our study with regard to the cost of surgery, a harmonic scalpel is a comparatively costlier modality than LigaSure. For this reason, we attempted to switch to using LigaSure for sutureless appendectomy.

LigaSure was found to have higher burst pressure than HS with faster cutting time and has been found effective and safe in preclinical studies [15]. de Souza et al. conducted studies on rabbits to find out the efficacy of LigaSure in appendectomy and compare it with simple ligature and conventional therapy. The group in which LigaSure was applied had fibrosis in 100% of animals. This technique induces enough fibrous tissue to obstruct the leakage of enteric content [16]. A study conducted by Helpman and Covens used the LigaSure device for the performance of an appendectomy during laparoscopic surgery for gynecologic malignancies in 14 patients. There were no conversions to laparotomy and no major intraoperative or postoperative complications [17]. Yavuz et al. studied 24 specimens of subtotal colectomy or right hemicolectomy and used a harmonic scalpel, conventional technique, and LigaSure for appendectomy. All three techniques were found to be equally safe [18].

The mean operative time for 53 appendectomies by LigaSure was 27.8 ± 6.72 minutes (range: 21-50 minutes). All operations were performed by the same surgeon with experience of more than 15 years in laparoscopic surgeries. We found out that our data was consistent with the other studies done using energy devices. We had earlier compared the sealing of the base of the appendix by harmonic scalpel (HS) and endoloop and observed significantly shorter operative time in the HS group of 28.46 ± 7.19 minutes (range: 17-48 minutes) versus 43.34 ± 6.7 minutes (range: 29-58 minutes), with no added complications [14]. Two similar studies that have been conducted in Egypt and Pakistan using HS have a mean operative time of 38.95 ± 3.55 and 31.6 ± 4.17 minutes, respectively, with no significant morbidity [19,20]. Khanna et al. utilized bipolar cautery for sealing the lumen of the appendix in 47 patients with a median duration of surgery of 25 minutes and a postoperative hospital stay of three days [21].

We observed a reduced operative time with respect to the use of ligature and various mechanical devices as cited in a recent Cochrane review [22]. We feel that applying a ligature, or even a mechanical device, will lead to an increase in the duration of surgery. LA by LigaSure has the potential to decrease the operative time as the mesoappendix and appendix can be dissected with a single device, and there is no need for the interchange of the instrument. The division of the mesoappendix can be done without vessel isolation, therefore making it possible to divide the mesoappendix rapidly without bleeding.

Our institute is a tertiary-level government hospital, and the cost of the performing LA by LigaSure device is INR 1500 or $19.7. The methods are covered in the national public health insurance scheme (Ayushman Bharat Yojana), which covers the cost of surgery. So, the surgery was performed irrespective of the financial status of the patient.

In some studies, concerns of lateral thermal damage of the appendicular stump due to heat dissipation have been raised; however, we do not find the lateral heat dissipation by LigaSure of any clinical relevance, and we did not observe any complication due to it [23].

A single case of surgical site infection occurred during our study. The small percentage of surgical site infections and no intra-abdominal abscess formation indicate that the technique for appendicular stump closure is safe. A single case of mild subcutaneous emphysema over the abdomen was observed, which was managed conservatively.

According to our study results, appendiceal stump closure by LigaSure is an acceptable laparoscopic procedure with encouraging intraoperative and postoperative results. The potential benefits perceived by the use of the LigaSure device include preventing spillage of the appendiceal contents into the peritoneal cavity as both the appendiceal stump and the severed appendix are closed. Tissue sealing and division can be achieved with one application of the LigaSure sealer/divider. The thermal effects are confined within the jaw, minimizing the probability of adjacent tissue injury. Tissues can be sealed and divided without dissection of the vessels lying within it. Consequently, the need for instrument interchange is reduced, thereby reducing the operative time. Because of these perceived advantages, the LigaSure system was found to be very helpful in performing a laparoscopic appendectomy.

Study limitations

This study is a single-arm non-randomized trial. As no previous study has been conducted on the usage of LigaSure during laparoscopic appendectomy, we were extra vigilant during our study. Complicated cases such as appendicular lump, appendicular abscess, and appendicular perforation were not included in our study. An alternate technique was used when the base of the appendix was found to be gangrenous or a rim of cecal tissue needed to be dissected along with the appendix. However, LigaSure was used irrespective of the diameter of the appendicular stump and even in cases of inflamed appendicular base and gangrene of the appendiceal tip. Further study is needed to find out the safety of LigaSure in complicated cases of appendicitis.

## Conclusions

Our study provides a small yet significant contribution to the debatable approaches to appendectomies and how to proceed with one. We demonstrated that LigaSure can be used confidently in laparoscopic appendectomy in handling the base of the appendix. The important benefits of it are nonusage of any suture or stapling device, ease of performing, precise dissection with fine jaw design, and insignificant thermal spread. Complications are minimal. It further demonstrates that there is room to believe that the method is safe and feasible; thus, there is a reason to do larger studies to further validate and evaluate this method of appendectomy.
